# Genomic insights into probiotic potential and metabolic adapability of food derived *Lactiplantibacillus plantarum* and *Pediococcus acidilactici*

**DOI:** 10.3389/fmicb.2026.1847858

**Published:** 2026-06-03

**Authors:** Tariq Aziz, Chasheen Fizza, Liqing Zhao, Zhennai Yang, Layla A. Alahmari, Fatma Alshehri, Ashwag Shami, Omniah A. Mansouri

**Affiliations:** 1 Department of Food Science and Technology, College of Chemistry and Environmental Engineering, Shenzhen University, Shenzhen, Guangdong, China; 2 School of Biomedical Engineering, Shenzhen University, Shenzhen, Guangdong, China; 3 Key Laboratory of Geriatric Nutrition and Health of Ministry of Education, Beijing Advanced Innovation Center for Food Nutrition and Human Health, Beijing Technology and Business University, Beijing, China; 4 Department of Community Health, College of Applied Medical Sciences, Northern Border University, Arar, Saudi Arabia; 5 Department of Biology, College of Science, Princess Nourah bint Abdulrahman University, Riyadh, Saudi Arabia; 6 Department of Biological Sciences, Collage of Science, University of Jeddah, Jeddah, Saudi Arabia

**Keywords:** comparative genomics, food biotechnology, lactic acid bacteria (LAB), functional annotation, metabolic plasticity

## Abstract

**Introduction:**

Probiotic lactic acid bacteria derived from agro-food sources play a pivotal role in promoting human health and advancing functional food development, however the genomic determinants underlying their adaptive versatility and probiotic functionality remain insufficiently characterized.

**Methodology:**

The current study employs a comparative genomics framework to evaluate the genetic diversity, metabolic plasticity, and evolutionary trajectories of *Lactiplantibacillus plantarum* (HMX2 and NMGL2) and *Pediococcus acidilactici* (BCB1H), aiming to elucidate the molecular determinants and functional mechanisms governing their probiotic efficacy. Various genomic analysis, including whole genome sequencing, genome annotation, average nucleotide identity (ANI), and Pan Genome analysis, was performed to assess the mechanism of adaptations and genomic variations.

**Results:**

NMGL2 exhibited the largest genome of 3.46 Mb among these strains, while having a total of 3,402 genes together with plasmids, which enhanced metabolic flexibility. A total of 59 genes were found which are likely linked with the functions of probiotic-related traits such as adhesion, immune modulation, and stress response. Although 59 genes were identified to be linked to probiotic characteristics like adhesion, immunomodulation, and stress resistance, the identified genomic features reflect the potential functionality and do not necessarily result in phenotypes.

**Discussion:**

The effectiveness of a probiotic is assessed based on more than just the availability of these genes; their regulation and expression are crucial. *L. plantarum* HMX2 and NMGL2 are found to be the same species but differ slightly at the genetic level, as indicated by the ANI between the two bacteria, which stands at 99.80%. On the other hand, the ANI between *P. acidilactici* BCB1H and *L. plantarum* is only around 68%, which is way less than 95% and thus indicates that they are not of different species. This shows that the two genera diverged at an early stage in their evolutionary path. Through Pan-Genome analysis, gene clusters were identified for varying levels of adaptability. The genetic features of *L. plantarum* NMGL2 suggest potential adaptability for industrial and probiotic use, though these predictions require experimental validation. The combined studies on transcriptomics and metablomics are required for the validation of functional potential in the genomics of these studies.

## 1 Introduction

Probiotics, particularly Lactic Acid Bacteria (LAB), have an important role in gut health as well as immunological and metabolic function. *Lactiplantibacillus plantarum* and *Pediococcus acidilactici* are well-known bacteria for their fermentation and antibacterial characteristics (Rastogi and Singh, 2022). As interest in probiotic-based functional foods and medicines has grown, genomic investigations are now required to identify appropriate strains possessing superior functional efficacy and robust safety profiles (Kaur et al., 2022). Comparative genomics approaches enable scientists to detect specific qualities in probiotic strains, allowing them to choose particular bacterial strains with increased prebiotic capabilities, higher adhesion abilities, and enhanced health-promoting attributes (Torres-Maravilla et al., 2022). Lactiplantibacillus and Pediococcus species are well-known for having health-improving features, such as strong antioxidants and the capability to sustain viability in the digestive system due to different protective strategies (Blazheva et al., 2022). In spite of the fact that probiotic and postbiotic capabilities of these genera have been discovered, the exact genome-based determinants of strains HMX2, NMGL2, and BCB1H should be revealed yet. Although scientists have recognized their potential as probiotics, they lack a thorough understanding of how their DNA influences their health-related properties. Several studies have explored shared probiotic qualities in various species, but a genomic comparison technique shows distinct genetic components that lead to their improved functional capacities (Peng et al., 2023). Identifying the genetic variations between these strains requires significant investigation to choose the most suited probiotic strain for human health purposes (da Silva et al., 2024). Comparative genomics helps scientists to find important indicators related to probiotics by analyzing genes that affect gut adhesion, stress tolerance, antimicrobial peptide synthesis, and glucose metabolism (Garcia-Gonzalez et al., 2022). The investigation of core and unique genes with functional pathways and metabolic features allows researchers to choose the probiotic strain with the best qualities. The adoptive assessment enables scientists to foresee security features using tests that evaluate for antibiotic resistance and virulence-related genes. Despite the availability of genetic data for numerous probiotic strains, there is no coordinated comparative research of *L. plantarum* HMX2, *L. plantarum* NMGL2, and *P. acidilactici* BCB1H (Zhang et al., 2024).

The current study aims to assess the genetic relevance of *L. plantarum* HMX2, *L. plantarum* NMGL2, and *P. acidilactici* BCB1H to identify the strain with the greatest health advantages. The work uses computational genomic approaches to discover functional genes and develop metabolic pathways, as well as to predict features appropriate for probiotic applications. The findings of this study will improve researchers’ capacity to choose ideal probiotic strains for food manufacture, microbial gut management, and medicinal benefits. This comparative genomic analysis approach can be used to understand the functional and health-related properties of *L. plantarum* HMX2, *L. plantarum* NMGL2, and *P. acidilactici* BCB1H to discover the optimal probiotic strain. These strains were selected due to their documented efficacy in food fermentation and preliminary evidence of their health-promoting properties (Aljohani et al., 2025; Zhang et al., 2025; Aziz et al., 2023b). To identify both conserved and distinct genetic determinants of probiotic adaptation, a cross-genus comparison between *Lactiplantibacillus* and *Pediococcus* was conducted. This study investigates genetic markers that detect prebiotic metabolism, as well as the capacity to stick to the gut, antibacterial characteristics, and immunological regulation, to build enhanced next-generation probiotics. Unlike previous studies that provided initial genomic descriptions for *L. plantarum* HMX2 (Aziz et al., 2024c), NMGL2 (Aziz et al., 2025), and *P. acidilactici* BCB1H (Hu et al., 2025), this study provides a comparative framework that connects their genomic composition with evolutionary divergence and probiotic functionality. The aim is to identify shared and strain-unique genes that underlie metabolic flexibility, environmental adaptation, and potential industrial relevance. Metatranscriptomics, in conjunction with metabolomic methods, will aid in defining the possible activities of these strains, allowing for their use in customized nutrition, biological food production, and gut health management.

## 2 Materials and methods

### 2.1 Bacterial strains selection

The *L. plantarum HMX2*, *L. plantarum NMGL2*, and *P. acidilactici BCB1H* strains were isolated from traditional fermented dairy products in China and maintained. The strains were grown in MRS de Man Rogosa Sharp medium at 37 °C for 20 h. These strains were separately stored at −80 °C with 50% glycerol before they could be revived through three successive cultivations in MRS medium at 37 °C for 20 h. The revival process involving 37 °C MRS medium culture for 12 h with subsequent two-generation subculture led to the use of these strains isolated from fermented dairy products for MRS medium inoculation at 37 °C for 20 h. Standard laboratory procedures involved preparing the strains for analysis through standard bacterial culturing and DNA extraction techniques (Hu et al., 2023; Sadanov et al., 2023).

### 2.2 DNA extraction and sequencing

Genomic DNA was extracted from *L. plantarum* HMX2, *L. plantarum* NMGL2, and *P. acidilactici* BCB1H through a bacterial genomic DNA extraction kit (Beijing Tiangen) based on the manufacturer’s protocol. DNA quality analysis was performed using a Nanodrop spectrophotometer and agarose gel electrophoresis. The genome of *L. plantarum* HMX2 was sequenced to 12 × coverage and assembled using ABySS v.12.1 into a 3,322,298 bp circular chromosome. Following assembly, functional annotation was performed via the NCBI Prokaryotic Genome Annotation Pipeline (PGAP), and the sequence was deposited under Accession No. GCF_025144505.1. Similarly, the genomic data for *L. plantarum* NMGL2 was submitted to NCBI under Accession No. GCA_044590115.1. The sequences of *P. acidilactici* BCB1H were generated through the Illumina NovaSeq 6,000 platform using TruSeq DNA PCR-Free Library Preparation Kit. Raw reads were assessed using FastQC v0.11.9, and low-quality bases/adapter sequences were trimmed with Trimmomatic v0.39 (Q20, minimum read length 50 bp). *De novo* assembly was performed using SPAdes v3.15.5 with k-mers 21, 33, 55, and 77. Assembly quality was evaluated with QUAST v5.2.0 (N50, number of contigs, GC content), and genome completeness/contamination was estimated with CheckM v1.2.2 and submitted to NCBI under Accession No. GCF_021568595.1. The *L. plantarum* NMGL2 was also submitted to NCBI under the Accession No. GCA_044590115.1 (Hu et al., 2023).

### 2.3 Genome assembly and annotation

The draft assemblies generated from SPAdes were used as input for annotation, and the genome annotation was carried out using Prokka v1.14.6^1^ (Seemann, 2014). The BV-BRC^2^ platform was used to cross-validate genome annotations produced by Prokka, specifically to validate gene predictions and functional assignments. Post-assembly quality metrics were evaluated using standard parameters (Olson et al., 2023).

### 2.4 Functional annotation and subsystem distribution

The Bacterial and Viral Bioinformatics Resource Center (BVBRC) (see text footnote 2) performed the functional annotation while conducting subsystem distribution analysis. The RASTtk pipeline, which is used in BV-BRC to assign annotated genes into functional subsystems, was used to perform subsystem coverage analysis. The overall subsystem coverage was determined by calculating the ratio of genes mapped to subsystems to those not assigned. The subsystem annotation process linked genes connected to biological processes into distinct groups, which included metabolic pathways as well as stress response elements and virulence factors. The research examined metabolic functions and adaptations between *Lactiplantibacillus plantarum* and *Pediococcus acidilactici* through functional subsystem analysis (Olson et al., 2023).

### 2.5 Genome visualization and GC composition analysis

Genome visualization and GC composition analysis served to understand the structure and composition elements of the genomes. The ProkSee^3^ software generated a visual display of circular genomes, which represented both coding sequences and rRNA and tRNA genes and GC content variations and displayed potential genomic features (Grant et al., 2023).

### 2.6 Average nucleotide identity (ANI) analysis

The Average Nucleotide Identity (ANI) procedure determined the genomic similarities between different strains. The ANIb algorithm (BLAST-based approach) was used to perform the ANI analysis in IPGA v1.09^4^. A 1,000 bp fragment window size and a 200 bp step size were used for pairwise genome comparisons. The alignment method used to determine sequence similarities was BLASTN, and the minimum alignment identity threshold was set at 70%. In order to determine taxonomic placement and genomic relatedness, these ANI values were then clustered and displayed as a heatmap. Heatmap clustering of ANI values was performed in IPGA v1.09 using the UPGMA algorithm with Euclidean distance as the similarity measure (Liu et al., 2022).

### 2.7 Phylogenetic analysis

Phylogenetic studies helped to establish evolutionary relationships between different genomes under investigation. The 16S rRNA gene sequences of the isolates were aligned using ClustalW for the Multiple Sequence Alignment (Larkin et al., 2007). The Neighbor-Joining method was used to build phylogenetic trees with the Kimura 2-parameter model in MEGA X and visualized in iTOL. The reliability of the inferred topology was assessed by evaluating branch support using 1,000 bootstrap replicates (Kumar et al., 2018; Letunic and Bork, 2021).

### 2.8 Pan-genome analysis

Pan-genome analysis investigated genomic diversity along with evolutionary relationships between the studied bacterial strains. The PanGenome analysis was performed using Roary v3.13.0 for the correct identification of core and accessory genes through complete gene classification. Ten additional reference genomes for *L. plantarum* and *P. acidilactici* were retrieved from the NCBI RefSeq database. These were selected based on their Complete assembly status and established status as type strains, providing a robust comparative framework for identifying unique genetic features in HMX2, NMGL2, and BCB1H. The shared genomic elements among all strains made up the core genes, whereas strain-specific and variably present genes formed the accessory genes section, which added to genetic diversity. The assessment of functional variation among the genomes was done by performing gene cluster analysis to determine orthologous groups. A pan-genome accumulation curve served to track gene discoveries through the addition of more genomes, which offered information about genomic flexibility as well as gene acquisition mechanisms and evolutionary patterns (Liu et al., 2022; Sun et al., 2020; Milani et al., 2017; Zhang et al., 2018).

### 2.9 Prediction of secondary metabolites

With the aim to identify Non-Ribosomal Peptide Synthetases (NRPS), Polyketide Synthases (PKS), bacteriocins, terpenes, and other cluster types, biosynthetic gene clusters (BGCs) were predicted using antiSMASH v6.0^5^, with default parameters and annotated.gbk files from Prokka as input. This analysis enabled the detection of gene clusters responsible for the production of various secondary metabolites, including antibiotics, siderophores, and other bioactive compounds. The identified clusters were classified based on their homology to known biosynthetic pathways, allowing for the prediction of potential novel metabolites. The results provided insights into the strain’s metabolic potential and its capability to produce biologically significant compounds, contributing to its ecological role and potential biotechnological applications (Blin et al., 2021).

### 2.10 Prediction of mobile genetic elements

The mobileOG-db database^6^ helped to determine and classify mobile genetic elements inside bacterial genomes. Through this analysis, experts could identify plasmids as well as transposons and insertion sequences, and integrative conjugative elements (ICEs) that promote horizontal gene transfer. The detected MGEs received specific functional classifications through which researchers enhanced their knowledge about genomic adaptability and antimicrobial resistance, and virulence properties. The analysis brought knowledge about genome evolutionary patterns while showing how genomes respond to changes across various environments (Brown et al., 2022).

## 3 Results

### 3.1 Genome annotation and key genomic features

Analysis of *L. plantarum* HMX2, *L. plantarum* NMGL2, and *P. acidilactici* BCB1H genomes through comparison reveals their substantial genome structural variations and overlaps. The genome of *L. plantarum* NMGL2 stands as the most extensive because it holds 3,458,350 bp compared to *L. plantarum* HMX2, 3,322,298 bp, and *P. acidilactici* BCB1H, 1,915,620 bp genome size. All strains show excellent genomic consistency, and *L. plantarum* NMGL2, along with *L. plantarum,* demonstrate complete genome integrity according to CheckM results. The genome of *L. plantarum* NMGL2 contains the greatest number of coding sequences at 3,402, but *P. acidilactici* BCB1H possesses only 1,905 sequences. The GC content within the genes of *L. plantarum* HMX2 and *L. plantarum* NMGL2 stays homogenous at 44.3–44.5% but BCB1H exhibits lower GC content at 42.37%. Two plasmids belong to *L. plantarum* NMGL2, which distinguishes it from *L. plantarum* HMX2, with an unknown plasmid characterization and BCB1H without documented plasmids. Major environmental pressures have likely triggered the development of 25 antibiotic resistance genes inside the bacteria *L. plantarum* NMGL2 and *L. plantarum* HMX2. *L. plantarum* NMGL2 shows a higher number of transporter and stress-related genes, suggesting potential adaptability. Genetic studies revealed that *L. plantarum* NMGL2 holds a complex genetic structure together with evolutionary adaptations, especially for food-rich environments, which links to its discovery in food collected from Beijing, China. Table 1 summarizes the key characteristics of all three strains under study.

**Table 1:** Key characteristics of the *L. plantarum* HMX2, *P. acidilactici* BCB1H, and *L. plantarum* NMGL2.

Feature	*L. plantarum* HMX2	*P. acidilactici* BCB1H	*L. plantarum* NMGL2
Genome length (bp)	3,322,298	1,915,620	3,458,350
Chromosomes	1	N/A (4 contigs)	1
Plasmids	Unknown	Unknown	2
Contigs	1	4	3
GC content (%)	44.51	42.37	44.34
tRNA count	65	56	81
rRNA count	16	12	16
CDS (Protein-coding genes)	3,242	1,905	3,402
Hypothetical proteins	1,424	379	1,456
Sequencing platform	Illumina HiSeq	Illumina HiSeq	Illumina HiSeq
Assembly method	ABySS v.12.1	ABySS v. MAY-2021	SOAPdenovo v.2.04
Genome quality	Good	Good	Good
CheckM completeness (%)	100	99.48	100
CheckM contamination (%)	N/A	0.85	0.2
Collection source	Fermented food (Sauerkraut)	Fermented food (Sauerkraut)	Food sample
Geographic location	China (Jilin Province)	China (Jilin Province)	China (Beijing)
Antibiotic resistance genes	25	23	25
Transporter genes	14	8	16
Isolation year	2021	2021	2020

### 3.2 Comparative functional annotation and subsystem distribution

**Figure 1:**
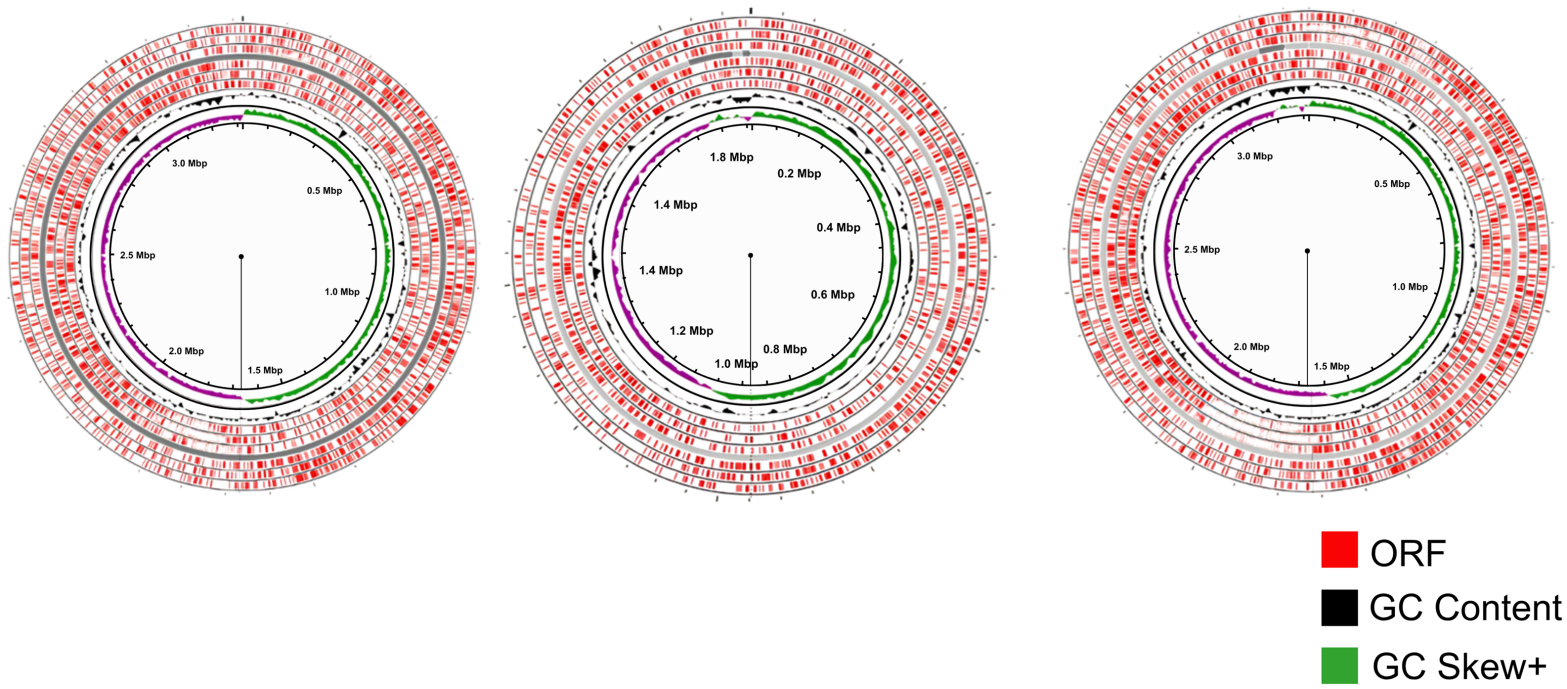
Circular genome maps of *L. plantarum* HMX2, *P. acidilactici* BCB1H, and *L. plantarum* NMGL2. The outermost rings represent open reading frames (ORFs) in red, while the inner rings display GC content (black), GC skew+ (green), and GC skew− (purple). Variations in GC content and skew indicate potential replication origins and strand-specific compositional asymmetries.

Subsystems distribution analysis distinguished the genomic features present in *L. plantarum* HMX2 from those of *L. plantarum* NMGL2 and *P. acidilactici* BCB1H. The three bacterial strains possessed a higher number of metabolic genes associated with metabolic subsystems, with *L. plantarum* NMGL2 (395 genes) and *L. plantarum* HMX2 (377 genes) having more metabolic genes compared to *P. acidilactici* BCB1H (267 genes). Protein processing genes achieved the highest enrichment in *P. acidilactici* BCB1H because this strain contained 197 representative genes for proteolytic activities. The highest number of stress response defense and virulence genes (85) appeared in *P. acidilactici* BCB1H because this strain showed higher representation of certain gene categories. The subsystem coverage analysis revealed that *P. acidilactici* BCB1H outperformed *L. plantarum* strains because of its higher number of annotated functional categories and functional potential. Figure 1 represents the comparative subsystem super class distribution of *L. plantarum* HMX2, *L. plantarum* NMGL2, and *P. acidilactici* BCB1H. The circular genome maps illustrate the proportion of genes associated with various functional subsystems, including metabolism, protein processing, stress response, DNA processing, and other cellular functions. The subsystem coverage percentages indicate the proportion of annotated versus non-annotated genes in each genome. The higher representation of certain gene categories in *P. acidilactici* BCB1H was primarily driven by an enrichment of genes involved in acid stress (e.g., operon for pH homeostasis), oxidative stress (e.g., sodA and cat), and heat shock response (e.g., dnaK, groEL, and grpE). These findings suggest that while all three strains possess probiotic potential, BCB1H may exhibit superior resilience to the environmental fluctuations encountered during gastrointestinal transit and food processing. The genomic analysis validated the existence of critical probiotic attributes in all strains, including the bsh gene that aids in bile resistance and the atp operon, which is vital in the gut. The strains also possess attributes that help in colonizing the host, like the mub and srtA genes.Table 2 depicts the identification of subsystems in the genomes under study. Key probiotic and adaptive genetic determinants identified in the genomes are given below in the Table 3.

**Table 2:** The comparative subsystem super class distribution of *L. plantarum* HMX2, *L. plantarum* NMGL2, and *P. acidilactici* BCB1H.

Category	*L. plantarum* HMX2	*P. acidilactici* BCB1H	*L. plantarum* NMGL2
Metabolism	86	46	86
Protein processing	97	95	99
Energy	15	13	15
Stress response, defense, virulence	22	22	22
DNA processing	8	6	8
Cell processing	16	17	16
RNA processing	12	14	12
Cell envelope	10	7	10
Membrane transport	6	9	6
Regulation & cell signaling	3	9	3
Miscellaneous	1	1	1

**Table 3:** Key probiotic and adaptive genetic determinants identified in the genomes.

Functional category	Gene/Operon identified	Predicted probiotic/Adaptive role
Acid & bile tolerance	*bsh*, *atpABCDEFG* operon	Facilitates bile salt detoxification and pH homeostasis during GI transit.
Adhesion & colonization	*mub*, *srtA*	Supports binding to the intestinal mucosa and host colonization.
Stress response	*dnaK*, *groEL*, *grpE*, *sodA*, *cat*	Provides resilience against heat, oxidative, and environmental stressors.
Antimicrobial activity	*Plantaricin EF/JK*, *Pediocin-related loci*	Supports pathogen inhibition and niche stabilization.

### 3.3 Genome visualization and GC content analysis

The circular genome visualization of *L. plantarum* HMX2 alongside *P. acidilactici* BCB1H and *L. plantarum* NMGL2 shows open reading frame (ORF) distributions and GC content as well as GC skew. A sliding window method (1,000 bp window, 500 bp step size) was used to calculate the GC content and GC skew. GC content was the proportion of guanine and cytosine in each window, and GC skew was defined as (G − C)/(G + C). Red marks depict densely clustered ORFs that span the genomes. The green colorations of GC content variations, together with purple and green colors, represent the areas of GC skew point. Observations show stable genomic patterns that serve adaptation functions, which contribute to metabolic diversity among these bacterial strains. Figure 1 represents the circular genome maps of *L. plantarum* HMX2, *P. acidilactici* BCB1H, and *L. plantarum* NMGL2.

### 3.4 Average nucleotide identity (ANI) analysis

**Figure 2:**
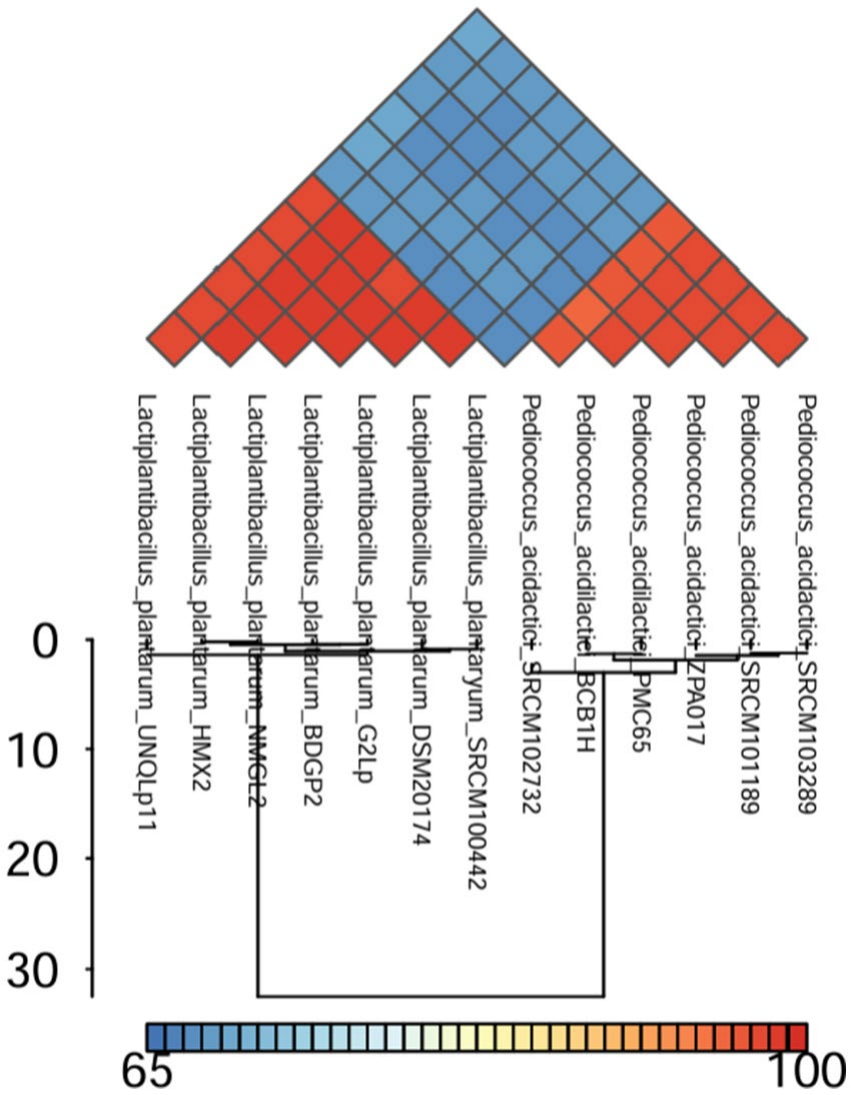
Heatmap representation of average nucleotide identity (ANI) values among *L. plantarum* and *P. acidilactici* strains. The color scale represents ANI percentages, with warmer colors indicating higher similarity and cooler colors indicating lower similarity. The results confirm a high degree of genomic similarity between *L. plantarum* strains and significant divergence from *P. acidilactici* BCB1H.

ANI values between *L. plantarum* HMX2 and *L. plantarum* NMGL2 and *P. acidilactici* BCB1H were obtained from the IPGA tool. The genome similarity of *L. plantarum* strains HMX2 and NMGL2 reached 99.80% based on their ANI values. The ANI values determined between *P. acidilactici* BCB1H and *L. plantarum* strains indicated a major genomic separation between these species through the observed values of ~68%. Specifically, the screening for stress response and defense mechanisms focused on four key physiological stressors: acid, osmotic, oxidative, and thermal stress. These were analyzed by filtering the BV-BRC Stress Response and Defense and Virulence subsystems to identify specific gene families associated with gastrointestinal survival and industrial robustness. The observed ANI of ~68% between *L. plantarum* and *P. acidilactici* provides a quantitative measure of the evolutionary distance between these two genera. This divergence serves as a baseline for the comparative analysis, highlighting the significance of the conserved genetic determinants identified in the subsequent pan-genome assessment. Figure 2 below depicts the Heatmap representation of Average Nucleotide Identity (ANI) values among *L. plantarum* and *P. acidilactici* strains. The detailed ANI values are given in Table 4.

**Table 4:** The comparative ANI values between *L. plantarum* HMX2, *L. plantarum* NMGL2, and *P. acidilactici* BCB1H.

Strain	*L. plantarum* HMX2	*L. plantarum* NMGL2	*P. acidilactici* BCB1H
*L. plantarum* HMX2	100	99.79557	68.09305
*L. plantarum* NMGL2	99.79557	100	68.28749
*P. acidilactici* BCB1H	68.09305	68.28749	100

### 3.5 Phylogenetic analysis and evolutionary relationships

**Figure 3:**
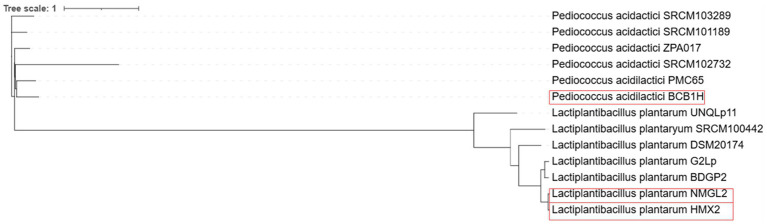
Phylogenetic tree depicting the evolutionary relationships among *L. plantarum* and *P. acidilactici* strains. The tree demonstrates a distinct clustering of *L. plantarum* and *P. acidilactici* species, highlighting their genetic divergence. The tree scale represents the evolutionary distance between the strains.

Phylogenetic analysis built a tree to explore the evolutionary comparisons between *Pediococcus acidilactici* strains along with *L. plantarum* strains. The phylogenetic tree reveals distinct species separation because *L. plantarum* strains maintain their genetic connection while grouping. Tests revealed that strains belonging to *P. acidilactici* created an evolutionary grouping that confirms their structural divergence from *L. plantarum*. The phylogenetic analysis produced results that backed up the ANI data to demonstrate that clear genetic differences exist between these two species. Figure 3 represents the phylogenetic tree depicting the evolutionary relationships among *L. plantarum* and *P. acidilactici* strains.

### 3.6 Pan-genome analysis and gene cluster distribution

**Figure 4:**
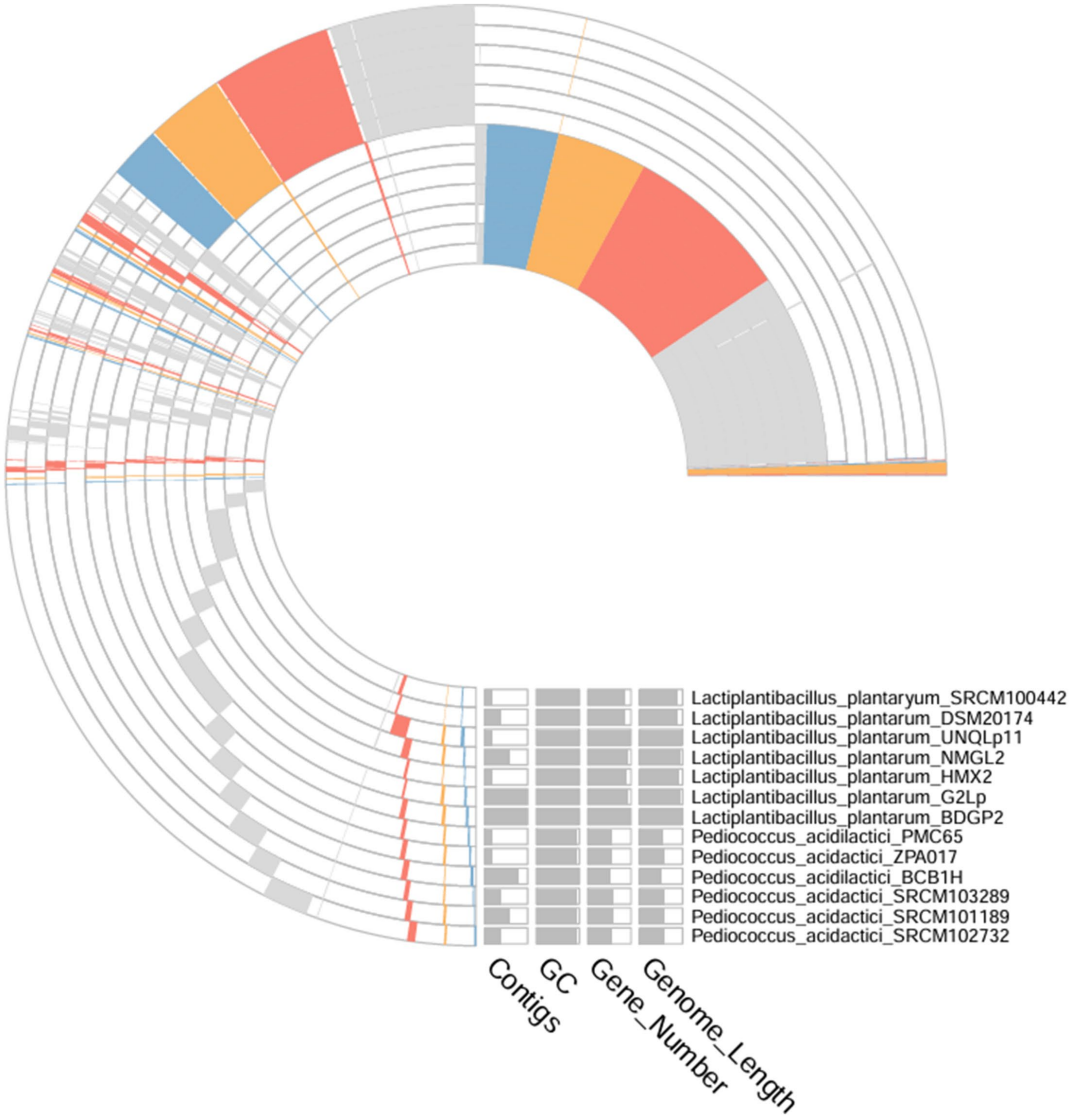
Circular comparative genomic representation of *L. plantarum* and *P. acidilactici* strains. The outer rings display gene presence-absence patterns, while the inner bars represent genome statistics, including contig number, GC content, gene count, and genome length. Differences in genomic composition highlight the genetic diversity between the two species.

IPGA PanGenome analysis was performed as a comparative framework to visualize overall genomic diversity and adaptation trends between *L. plantarum* and *P. acidilactici* strains, rather than as a unified species-level analysis. The results reflect shared conserved genes and distinct strain-specific features, highlighting intergenic divergence within lactic acid bacteria. Different genes associated with individual strains indicated distinct metabolic abilities as well as potential adaptations to unique niches. The pan-genome analysis showed a flexible open pan-genome structure because bacterial lineages keep acquiring new genes and evolving genetically. Figure 4 depicts the circular comparative genomic representation of *L. plantarum* and *P. acidilactici* strains.

**Figure 5:**
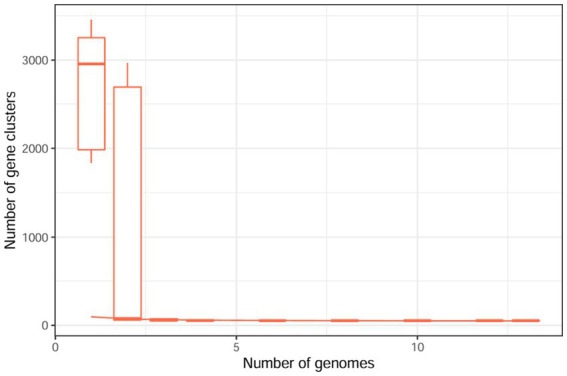
Pan-genome analysis of 13 *L. plantarum* and *P. acidilactici* strains using Roary. The plot shows the number of gene clusters identified as genomes are sequentially added, highlighting a large core genome and a smaller set of accessory genes.

The analysis of core gene clusters conserved across all strains identified well-conserved genetic sequences common to all investigated strains. A defined clustering approach identified clusters containing essential gene sets found in all examined genomes. The core genome contains genes that facilitate crucial biological processes needed for survival and metabolic operations for these organisms. Study findings demonstrate that certain genomic regions show a high degree of conservation across the strains in genomic areas that maintain core biological activities. Several accessory gene clusters, together with strain-specific gene clusters, demonstrate the adaptive potentials and ecological flexibility of these strains. Figure 5 represents the distribution of gene clusters across the analyzed genomes.

**Figure 6:**
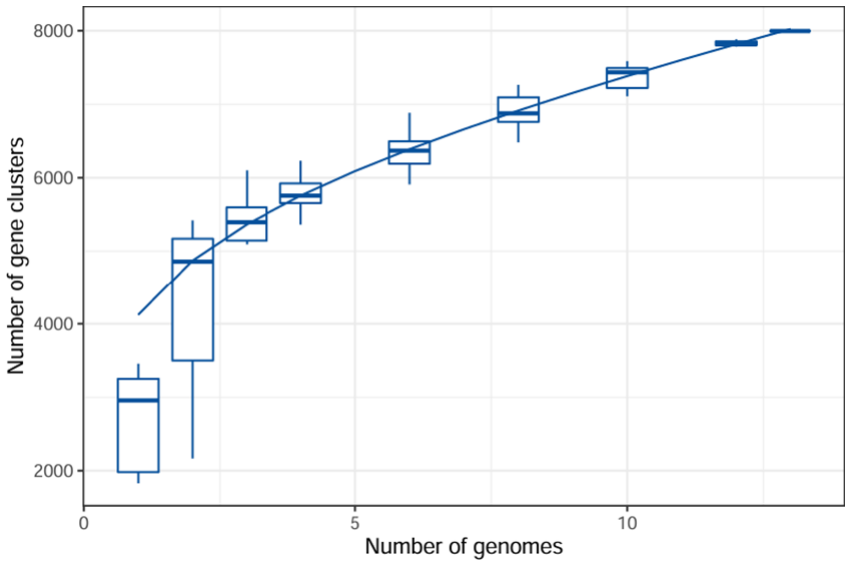
Pan-genome accumulation curve displaying the number of gene clusters as a function of the number of genomes analyzed. Box plots represent variability in gene cluster counts, while the fitted curve suggests an open pan-genome model.

The analysis of selected *L. plantarum* and *P. acidilactici* strain genomes reveals that gene cluster numbers steadily grow with each added genome sequence. A relatively open pan-genome structure exists due to genomic differences between bacterial strains. Different box plot results show that gene cluster accumulation displays reduced variability when utilizing additional genomes. Figure 6 depicts the pan-genome accumulation curve displaying the number of gene clusters as a function of the number of genomes analyzed.

**Figure 7:**
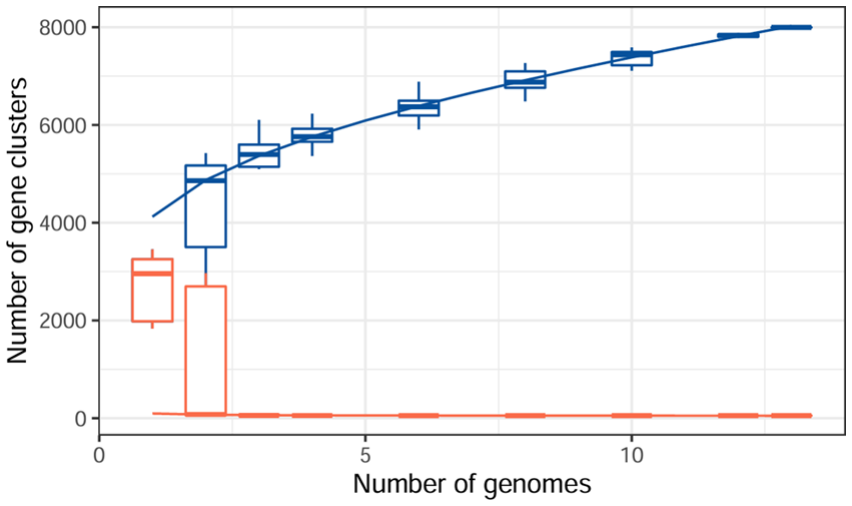
Pangenome accumulation curve of *P. acidilactici* and *L. plantarum*. The blue curve represents the core genome, showing the number of conserved gene clusters as more genomes are added. The red curve represents the accessory genome, displaying the number of variable gene clusters presents across different strains. Boxplots indicate the variation in gene counts at each genome addition step.

The considerable genetic distance that exists between the two genera is confirmed statistically using the Average Nucleotide Identity (ANI), which comes to around 68%. The number of core gene clusters present in the pangenome was found to be 476, accounting for about 14 to 25% of each genome. In the pangenome, the accessory genome occupies most of the space. The pangenome accumulation curve (Figure 6) reflects an open pangenome model, as confirmed by Heap’s Law metrics. While the total number of gene clusters increases with genomic expansion, the core genome reaches a plateau, suggesting that the true core pangenome is a highly conserved set of essential genes required for the survival and metabolic integrity of these lactic acid bacteria. Figure 7 depicts the pangenome accumulation curve of *P. acidilactici* and *L. plantarum*. The blue curve represents the core genome, showing the number of conserved gene clusters as more genomes are added. Since *L. plantarum* and *P. acidilactici* belong to different genera, the observed open pangenome trend should be interpreted as a representation of combined genetic diversity across probiotic LAB, not as a true species-level pangenome structure.

**Figure 8:**
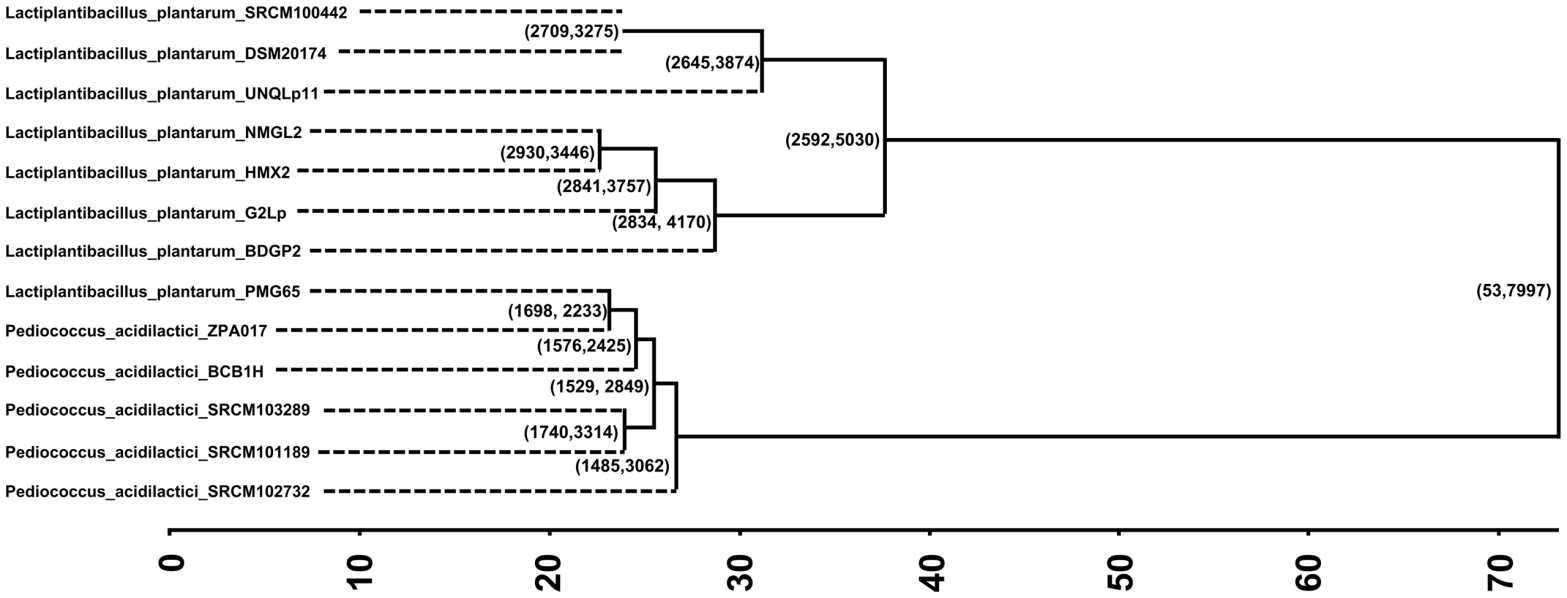
Dendrogram depicting hierarchical clustering of *L. plantarum* and *P. acidilactici* strains based on shared gene content. Branch lengths represent genomic differences, highlighting interspecies divergence and intraspecies variability.

The hierarchical clustering analysis splits *Lactiplantibacillus plantarum* apart from *Pediococcus acidilactici* in separate clades because of their distinct genomic differences. The gene content analysis shows distinct levels of variations within each species existing through separate strain subclusters. An extensive genetic distance between the genera shows substantial variations exist in their fundamental genetic components and extra genetic material. The Figure 8 depicts the dendrogram depicting hierarchical clustering of *Lactiplantibacillus plantarum* and *Pediococcus acidilactici* strains based on shared gene content.

**Figure 9:**
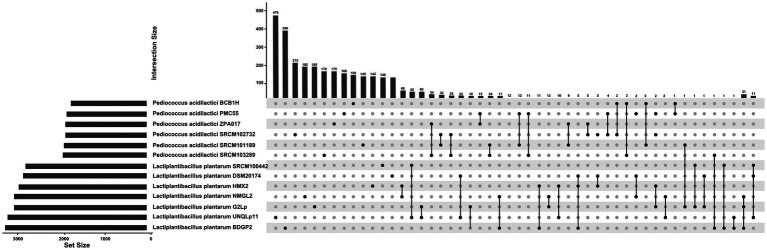
UpSet plot illustrating the shared and unique gene clusters among *P. acidilactici* and *L. plantarum* strains. The left bar plot represents the total gene clusters per strain, while the upper bar chart shows the number of gene clusters shared among strain combinations. The dots and connecting lines indicate intersecting sets of strains with shared genes.

The UpSet plot displays the arrangement of gene clusters that are shared with clusters existing only in *P. acidilactici* and *L. plantarum* strains. The left portion displays strain-specific gene quantities, whereas the upper bar graph depicts the size distribution of gene intersections between various strain pairs. The largest set of shared gene clusters (476) appears among different strains, which demonstrates a basic group of essential genes common to these microbial species. The accessory genes plus strain-specific genes appear as smaller intersections in the data, which demonstrates genomic variations across species, along with resulting specific portions in each species. It appears from the gene distribution that differentiation happens at the species level while specific adaptations emerge at the strain level. Figure 9 depicts the UpSet plot illustrating the shared and unique gene clusters among *P. acidilactici* and *L. plantarum* strains.

### 3.7 Prediction of secondary metabolites

**Figure 10:**
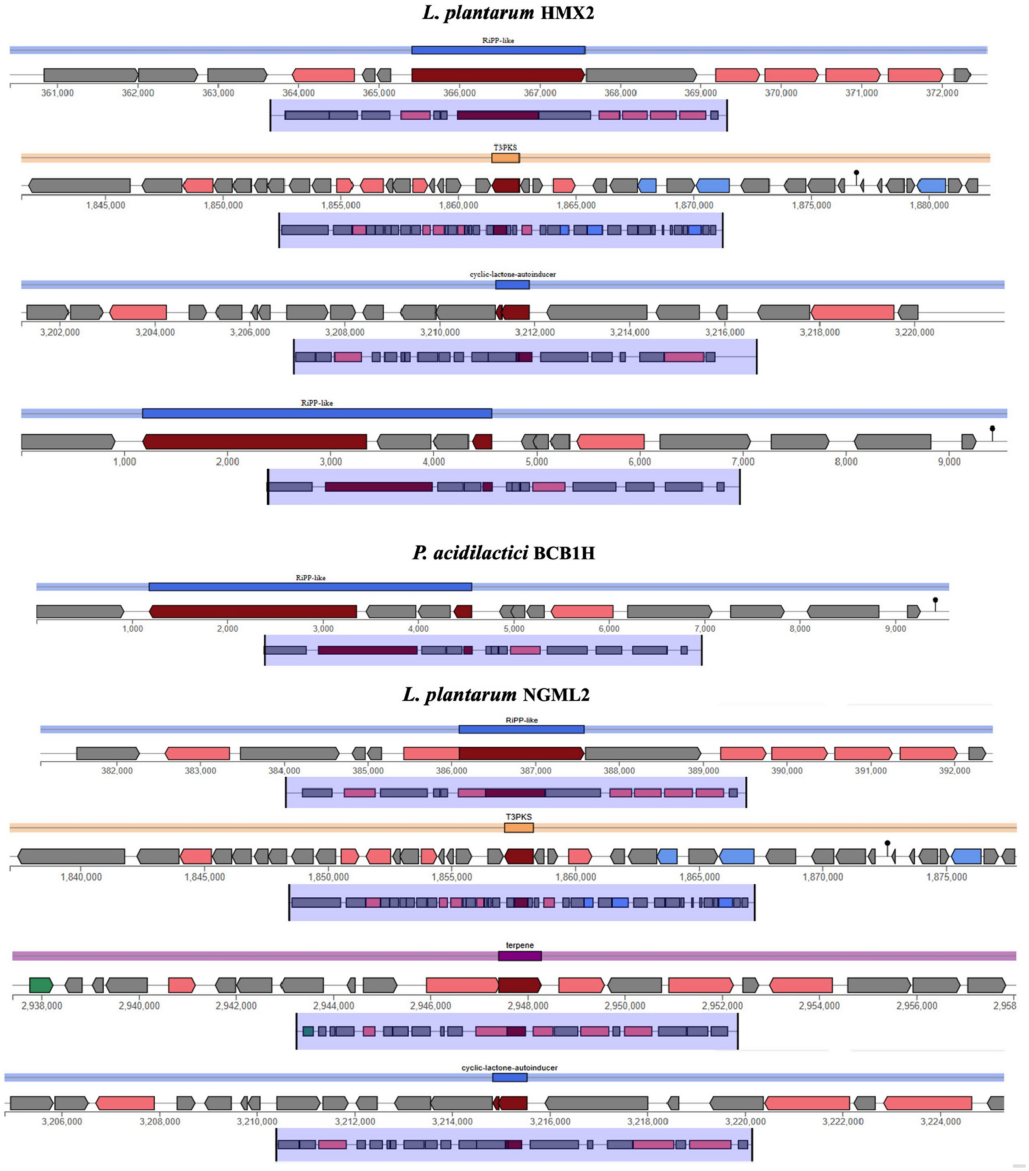
Prediction of biosynthetic gene clusters (BGCs) in the genome using antiSMASH. The identified clusters, HMX2, BCB1H, and NMGL2, contain genes encoding secondary metabolite biosynthesis. Red and pink represent core biosynthetic genes, gray indicates flanking genes, and blue bars denote predicted cluster boundaries. Identified pathways include terpenes, polyketides, and NRPS-related metabolites, suggesting their potential role in bioactive compound synthesis.

Analysis of BGCs revealed specific probiotic determinants, including Plantaricin EF/JK in *L. plantarum* and Pediocin PA-1 homologs in *P. acidilactici* BCB1H, which are key for pathogen inhibition. Additionally, the presence of terpene and T3PKS clusters suggests potential antioxidant and signaling roles, highlighting the functional basis for the strain’s health-promoting effects. The analyzed biosynthetic gene clusters, grouped into three main clusters called HMX2 and BCB1H together with NMGL2, contain fundamental biosynthetic genes as well as transporters and regulatory elements. The identified clusters show a broad spectrum of encoded secondary metabolites that incorporate terpenes and Polyketide Synthases (PKS) and Non-Ribosomal Peptide Synthases (NRPS) and hybrid pathways because these clusters demonstrate potential roles in antimicrobial and bioactive compound production. Key biosynthetic genes in the genomic regions appear as red/pink, while flanking genes are shown as gray, and predicted cluster boundaries appear as blue bars. *P. acidilactici* BCB1H contained a single RIPP-like BGC. *L. plantarum* NMGL2 carried four BGCs, including RIPP-like, T3PKS, terpene, and cyclic-lactone-autoinducer. These clusters are predicted to encode metabolites such as antimicrobial peptides, polyketides, terpenoids, and quorum-sensing molecules, highlighting strain-specific secondary metabolite potential. Figure 10 shows the predicted biosynthetic gene clusters (BGCs) in the genomes of HMX2, BCB1H, and NMGL2, highlighting core biosynthetic genes (red/pink), flanking genes (gray), and cluster boundaries (blue), with identified pathways including terpenes, polyketides, and NRPS-related metabolites, suggesting potential roles in bioactive compound synthesis.

### 3.8 Prediction of mobile genetic elements

**Figure 11:**
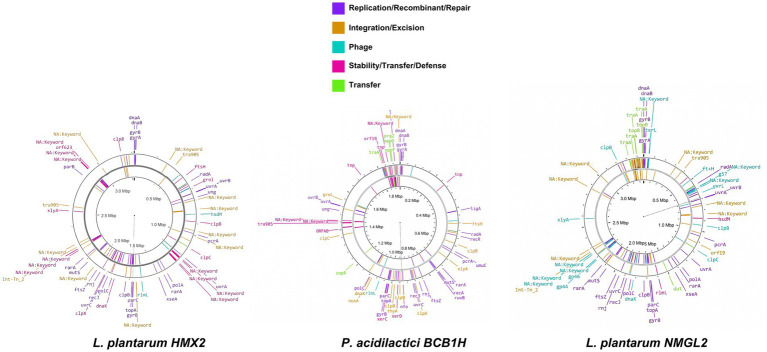
Prediction of mobile genetic elements (MGEs) in the genomes of *P. acidilactici* strain BCB1H, *L. plantarum* strain HMX2, and *L. plantarum* strain NMGL2. The figure illustrates the distribution and types of MGEs identified using mobileOG-db, highlighting strain-specific variations and their potential role in horizontal gene transfer and genomic adaptation.

MobileOG-db identified multiple Mobile Genetic Elements (MGEs) such as plasmids and transposons, and insertion sequences during the predictions made on three microbial genomes consisting of *Pediococcus acidilactici* strain BCB1H, *Lactiplantibacillus plantarum* strain HMX2, and *Lactiplantibacillus plantarum* strain NMGL2. The three genomes contained these elements, yet their number and arrangement displayed differences between strains. The study results indicate that horizontal gene transfer capabilities and genome adaptability may enhance the adaptive behavior as well as functional richness in these strains. Research data demonstrates that MGE presence contributes to the adaptive nature and ecological flexibility of analyzed organisms during evolutionary processes. MobileOG-db detected several MGEs, such as plasmids, insertion sequences, and transposons, in all three strains (*P. acidilactici* BCB1H, *L. plantarum* HMX2, and NMGL2). A number of these elements carried genes linked to stress (clp, groEL) and antibiotic resistance (tetM, ermB), underscoring their function in environmental adaptation and resistance spread. Therefore, the existence of MGEs shows that horizontal gene transfer contributes to functional diversity and pan-genome variability. Figure 11 illustrates the distribution and types of mobile genetic elements (MGEs) in the genomes of *P. acidilactici* BCB1H and *L. plantarum* strains HMX2 and NMGL2, highlighting strain-specific variations and their potential roles in horizontal gene transfer and genomic adaptation.

## 4 Discussion

Significant variations in genome organisation and functional capacity were found when *L. plantarum* HMX2, *L. plantarum* NMGL2, and *P. acidilactici* BCB1H were compared genomically. Of the three, NMGL2 had the most genes (3,402) and the largest genome (3.46 Mb). *P. acidilactici* BCB1H showed a lower value of 42.37%, whereas strains of *L. plantarum* consistently maintained a GC content of 44.3%. NMGL2, which carried two plasmids not found in the other strains, was further identified by plasmid profiling. While *P. acidilactici* and *L. plantarum* isolates had low ANI (~68%), which indicated a distant evolutionary relationship well below the 95% species-level threshold, the isolates’ high ANI (99.8%) confirmed their close relatedness (Min et al., 2022; Aziz et al., 2020). Genome visualization tools exhibited continual adaptation patterns and an open genome structure through pan-genome analysis, along with core and strain-specific genes that increase ecological adaptability (Zheng et al., 2020; Aziz et al., 2024a; Aziz et al., 2021).

Comparing *L. plantarum* and *P. acidilactici* at the genus level revealed variations in adaptation and evolutionary approach. *L. plantarum* NMGL2’s larger genome and plasmid complement demonstrate metabolic flexibility, allowing for niche-specific adaptation. The evolutionary pressures on *L. plantarum* NMGL2 favored metabolic flexibility primarily through its broad functional diversity and plasmid content, which likely provide greater genomic capacity for environmental adaptation, rather than being solely influenced by its GC content. Pangenomic investigations suggest an open pan-genome architecture, allowing these strains to receive extra genetic material for adapting to their environment and living (Yu et al., 2020; Aziz et al., 2023a). Roary provides a combined pan-genome analysis, while genus-wise separation was examined through IPGA-based UpSet plots. This allowed interpretation of core and accessory genome differences within each genus.

The findings on *L. plantarum* NMGL2 have substantial implications for both industrial and probiotic uses. *L. plantarum* NMGL2’s genomic features indicate potential for diverse metabolic functions that could be explored in future probiotic or fermentation studies. As highlighted in the results of the functional annotation analysis, the existence of bsh, atp operons, and mub genes is indicative of the strains’ ability to withstand gastrointestinal passage and adhere to the host mucosa. The results conform to previous genomic characterizations of high-performing probiotics Lactiplantibacillus and Pediococcus strains. Through its open pan-genome structure, *L. plantarum* NMGL2 continues to show potential for ongoing gene acquisition that enables it to tolerate environmental stressors more effectively to stressors and maintain metabolic activity across varied conditions across many habitats (Zhao et al., 2023; Aziz et al., 2024b). The pangenomic architecture present among these strains is characterized by high inter-generic variation and the continuous ability to acquire genes. Whereas *L. plantarum* NMGL2 displays better metabolic versatility owing to its large genomic size and distinct plasmids, the genomic design adopted by *P. acidilactici* BCB1H is more compact and emphasizes stress tolerance. This implies that although these lactic acid bacteria are both probiotic, they have evolved along different pathways owing to the various conditions under which they were collected from fermentation products and the environment (Yao et al., 2022; Aziz et al., 2023c; Aziz et al., 2023b).

The study provides important information regarding the genetic makeup of *L. plantarum* NMGL2, *L. plantarum* HMX2, and *P. acidilactici* BCB1H, but its findings must be interpreted under specific limits. Validation through laboratory testing is necessary to provide correct functional predictions, as relying entirely on computational genome analysis might create inaccuracies (Quach et al., 2024). Key probiotic-related genes, such as Hsp., GroEL, DnaK, Clp proteases (stress tolerance), bile salt hydrolase (BSH) and F₀F₁-ATPase (acid and bile resistance), mucus-binding proteins, sortase-dependent LPxTG proteins, and EPS biosynthesis clusters (adhesion), as well as bacteriocin, PKS, and NRPS gene clusters (antimicrobial activity), are present in the genomes of the strains under analysis. Together, these genetic traits promote colonisation, pathogen inhibition, host interaction, and gastrointestinal tract survival. Despite its usefulness, the study lacks examination of phenotypic traits for these strains when exposed to different environmental factors that impact their potential uses either as probiotics or industrially (Cheruvari and Kammara, 2024). To validate evolutionary trends among *L. plantarum’s* open pan-genome structure, more comparative data must be collected. Future study using combined transcriptome and metabolomic techniques might yield more extensive functional data. Additional study is required to assess the expected functional features of *L. plantarum* NMGL2, *L. plantarum* HMX2, and *P. acidilactici* BCB1H, utilizing both laboratory-based *in vitro* and animal-based *in vivo* testing. A broader comparative genomic investigation of other strains will improve our understanding of their natural evolutionary changes. Studies that combine transcriptome and metabolomic analysis will provide a more complete knowledge of the probiotic effect and industrial applications of these strains.

Crucially, all three strains had genetic determinants linked to probiotics. These include bile salt hydrolase (BSH) and F₀F₁-ATPase (acid and bile resistance), mucus-binding proteins and sortase-dependent LPxTG proteins (adhesion), exopolysaccharide biosynthesis clusters (colonisation), bacteriocin, PKS, and NRPS clusters (antimicrobial activity), and chaperones (Hsp., GroEL, and DnaK) and Clp proteases (stress tolerance). These characteristics work together to promote pathogen inhibition, host interaction, and gut survival.

The novelty of this work lies in integrating functional annotation and pan-genome analysis to uncover adaptive signatures across *L. plantarum* and *P. acidilactici*. While previous studies characterized these genomes individually, this comparative approach reveals broader patterns of genomic evolution, core functional conservation, and differential probiotic traits among related LAB strains. These interpretations are based on genome-level predictions and require validation through transcriptomic, proteomic, and phenotypic analyses. While there have been numerous findings based on this study, the major limitation that is associated with the study is its dependence on in silico prediction. Even though genomic sequences can provide the basis of probiotic activity within an organism, it is imperative for future studies to incorporate transcriptomics and metabolomics to validate these findings.

## 5 Conclusion

This comparative genomic study of *Lactiplantibacillus plantarum* HMX2, *L. plantarum* NMGL2, and *Pediococcus acidilactici* BCB1H reveals distinct patterns of genetic diversity, conserved core functions, and strain-specific adaptations. The identified genes related to stress tolerance, adhesion, and antimicrobial compound synthesis suggest possible roles in probiotic activity and environmental resilience. Collectively, these insights offer a genomic foundation for understanding how lactic acid bacteria adapt to different ecological and industrial niches. Nonetheless, since the conclusions rely solely on computational predictions, further transcriptomic, proteomic, and phenotypic investigations are required to substantiate the proposed functional roles and evaluate their practical relevance in probiotic and biotechnological applications.

## Data Availability

The datasets presented in this study can be found in online repositories. The names of the repository/repositories and accession number(s) can be found below: NCBI GenBank: GCF_025144505.1 (*L. plantarum* HMX2), GCA_044590115.1 (*L. plantarum* NMGL2), and GCF_021568595.1 (*P. acidilactici* BCB1H).

## Footnotes

1
https://github.com/tseemann/prokka

2
https://www.bv-brc.org

3
https://proksee.ca

4
https://nmdc.cn/ipga

5
https://antismash.secondarymetabolites.org

6
https://github.com/clb21565/mobileOG-db

## References

[ref1] Aljohani A. Rashwan N. Vasani S. Alkhawashki A. Tong Tong W. Xingyi L. . (2025). The health benefits of probiotic *Lactiplantibacillus plantarum*: a systematic review and meta-analysis. Probiotics Antimicrob. Proteins 17, 3358–3377. doi: 10.1007/s12602-024-10287-3, 38816672 PMC12532744

[ref2] Aziz T. Naveed M. Jabeen K. Shabbir M. A. Sarwar A. Zhennai Y. . (2023a). Integrated genome based evaluation of safety and probiotic characteristics of *Lactiplantibacillus plantarum* YW11 isolated from Tibetan kefir. Front. Microbiol. 14:1157615. doi: 10.3389/fmicb.2023.1157615, 37152722 PMC10158936

[ref3] Aziz T. Naveed M. Shabbir M. A. Khan A. A. Sarwar A. Haq T. U. . (2024a). Assessment of the whole genome sequencing of *Lactiplantibacillus plantarum* 13-3 for elucidation of novel bacteriocin producing gene cluster and confirmation of its potential probiotic functionality and safety applications. CyTA-J. Food 22:2329753. doi: 10.1080/19476337.2024.2329753

[ref4] Aziz T. Naveed M. Shabbir M. A. Sarwar A. Khan A. A. Hasnain A. . (2024b). Whole genome analysis of Tibetan kefir-derived *Lactiplantibacillus plantarum* 12-3 elucidates its genomic architecture, antimicrobial and drug resistance, potential probiotic functionality and safety. Front. Biosci. Landmark 29:147. doi: 10.31083/j.fbl2904147 38682181

[ref5] Aziz T. Naveed M. Shabbir M. A. Sarwar A. Khan A. A. Zhennai Y. . (2023b). Comparative genomics of food-derived probiotic *Lactiplantibacillus plantarum* K25 reveals its hidden potential, compactness, and efficiency. Front. Microbiol. 14:1214478. doi: 10.3389/fmicb.2023.1214478, 37455721 PMC10346846

[ref6] Aziz T. Naveed M. Shabbir M. A. Sarwar A. Naseeb J. Zhao L. . (2024c). Unveiling the whole genomic features and potential probiotic characteristics of novel *Lactiplantibacillus plantarum* HMX2. Front. Microbiol. 15:1504625. doi: 10.3389/fmicb.2024.1504625, 39611087 PMC11602494

[ref7] Aziz T. Sarwar A. Din J. u. Al Dalali S. Khan A. A. Din Z. U. . (2021). Biotransformation of linoleic acid into different metabolites by food derived *Lactobacillus plantarum* 12-3 and in silico characterization of relevant reactions. Food Res. Int. 147:110470. doi: 10.1016/j.foodres.2021.110470, 34399468

[ref8] Aziz T. Sarwar A. Fahim M. Din J. U. Al Dalali S. Din Z. U. . (2020). Dose-dependent production of linoleic acid analogues in food derived *Lactobacillus plantarum* K25 and in silico characterization of relevant reactions. Acta Biochim. Pol. 67, 123–129. doi: 10.18388/abp.2020_5167, 32187238

[ref9] Aziz T. Shabbir M. A. Sarwar A. Khan A. A. Zhao L. Yang Z. . (2025). Exploring the multifaceted probiotic potential of *Lactiplantibacillus plantarum* NMGL2, investigating its antimicrobial resistance profiles and bacteriocin production. J. Microbiol. Methods 236:107178. doi: 10.1016/j.mimet.2025.107178, 40543402

[ref10] Aziz T. Xingyu H. Sarwar A. Naveed M. Shabbir M. A. Khan A. A. . (2023c). Assessing the probiotic potential, antioxidant, and antibacterial activities of oat and soy milk fermented with *Lactiplantibacillus plantarum* strains isolated from Tibetan kefir. Front. Microbiol. 14:1265188. doi: 10.3389/fmicb.2023.1265188, 37817753 PMC10560984

[ref11] Blazheva D. Mihaylova D. Averina O. V. Slavchev A. Brazkova M. Poluektova E. U. . (2022). Antioxidant potential of probiotics and postbiotics: a biotechnological approach to improving their stability. Russ. J. Genet. 58, 1036–1050. doi: 10.1134/S1022795422090058

[ref12] Blin K. Shaw S. Kloosterman A. M. Charlop-Powers Z. Van Wezel G. P. Medema M. H. . (2021). antiSMASH 6.0: improving cluster detection and comparison capabilities. Nucleic Acids Res. 49, W29–W35. doi: 10.1093/nar/gkab335, 33978755 PMC8262755

[ref13] Brown C. L. Mullet J. Hindi F. Stoll J. E. Gupta S. Choi M. . (2022). mobileOG-db: a manually curated database of protein families mediating the life cycle of bacterial mobile genetic elements. Appl. Environ. Microbiol. 88, e00991–e00922. doi: 10.1128/aem.00991-22 36036594 PMC9499024

[ref14] Cheruvari A. Kammara R. (2024). Genomic characterization and probiotic properties of Lactiplantibacillus pentosus isolated from fermented rice. Probiotics Antimicrob. Proteins 17, 4442–4464. doi: 10.1007/s12602-024-10378-1, 39433653

[ref15] da Silva T. F. de Assis Glória R. Ferrary Americo M. dos Santos Freitas A. de Jesus L. C. L. Alvarenga Lima Barroso F. . (2024). Unlocking the potential of probiotics: a comprehensive review on research, production, and regulation of probiotics. Probiotics Antimicrob. Proteins 16, 1687–1723. doi: 10.1007/s12602-024-10247-x 38539008

[ref16] Garcia-Gonzalez N. Bottacini F. Van Sinderen D. Gahan C. G. M. Corsetti A. (2022). Comparative genomics of *Lactiplantibacillus plantarum*: insights into probiotic markers in strains isolated from the human gastrointestinal tract and fermented foods. Front. Microbiol. 13:854266. doi: 10.3389/fmicb.2022.854266, 35663852 PMC9159523

[ref17] Grant J. R. Enns E. Marinier E. Mandal A. Herman E. K. Chen C.-y. . (2023). Proksee: in-depth characterization and visualization of bacterial genomes. Nucleic Acids Res. 51, W484–W492. doi: 10.1093/nar/gkad326, 37140037 PMC10320063

[ref18] Hu G. Naveed M. Shabbir M. A. Sarwar A. Yousaf J. Zhennai Y. . (2025). Revolutionizing the probiotic functionality, biochemical activity, antibiotic resistance and specialty genes of *Pediococcus acidilactici* BCB1H via in-vitro and in-silico approaches. Zeitschrift für Naturforschung C 80, 103–118. doi: 10.1515/znc-2024-0074, 39026396

[ref19] Hu G. Wang Y. Xue R. Liu T. Zhou Z. Yang Z. (2023). Effects of the exopolysaccharide from *Lactiplantibacillus plantarum* HMX2 on the growth performance, immune response, and intestinal microbiota of juvenile turbot, *Scophthalmus maximus*. Foods 12:2051. doi: 10.3390/foods12102051, 37238869 PMC10217481

[ref20] Kaur H. Kaur G. Ali S. A. (2022). Dairy-based probiotic-fermented functional foods: an update on their health-promoting properties. Fermentation 8:425. doi: 10.3390/fermentation8090425

[ref21] Kumar S. Stecher G. Li M. Knyaz C. Tamura K. (2018). MEGA X: molecular evolutionary genetics analysis across computing platforms. Mol. Biol. Evol. 35, 1547–1549. doi: 10.1093/molbev/msy096, 29722887 PMC5967553

[ref22] Larkin M. A. Blackshields G. Brown N. P. Chenna R. McGettigan P. A. McWilliam H. . (2007). Clustal W and Clustal X version 2.0. Bioinformatics 23, 2947–2948. doi: 10.1093/bioinformatics/btm404, 17846036

[ref23] Letunic I. Bork P. (2021). Interactive tree of life (iTOL) v5: an online tool for phylogenetic tree display and annotation. Nucleic Acids Res. 49, W293–W296. doi: 10.1093/nar/gkab301, 33885785 PMC8265157

[ref24] Liu D. Zhang Y. Fan G. Sun D. Zhang X. Yu Z. . (2022). IPGA: a handy integrated prokaryotes genome and pan-genome analysis web service. iMeta 1:e55. doi: 10.1002/imt2.55, 38867900 PMC10989949

[ref25] Milani C. Duranti S. Bottacini F. Casey E. Turroni F. Mahony J. . (2017). The first microbial colonizers of the human gut: composition, activities, and health implications of the infant gut microbiota. Microbiol. Mol. Biol. Rev. 81. doi: 10.1128/mmbr.00036-17 PMC5706746 29118049

[ref26] Min B. Kwon Y.-J. Park S.-Y. Lim J. H. Shin C. H. Kim B.-K. . (2022). Genomic characteristics and comparative genomic analysis of a probiotic bacterial strain, Lactiplantibacillus plantarum CKDB008. Food Supp. Biomaterials Health 2. doi: 10.52361/fsbh.2022.2.e32

[ref27] Olson R. D. Assaf R. Brettin T. Conrad N. Cucinell C. Davis J. J. . (2023). Introducing the bacterial and viral bioinformatics resource center (BV-BRC): a resource combining PATRIC, IRD and ViPR. Nucleic Acids Res. 51, D678–D689. doi: 10.1093/nar/gkac1003 36350631 PMC9825582

[ref28] Peng X. Ed-Dra A. Yue M. (2023). Whole genome sequencing for the risk assessment of probiotic lactic acid bacteria. Crit. Rev. Food Sci. Nutr. 63, 11244–11262. doi: 10.1080/10408398.2022.2087174, 35694810

[ref29] Quach N. T. Nguyen T. T. A. Nguyen T. H. N. V. Nguyen T. T. N. Tran X. K. Chu N. H. . (2024). New insight into protective effect against oxidative stress and biosynthesis of exopolysaccharides produced by Lacticaseibacillus paracasei NC4 from fermented eggplant. Curr. Genet. 70:7. doi: 10.1007/s00294-024-01292-8 38743270

[ref30] Rastogi S. Singh A. (2022). Gut microbiome and human health: exploring how the probiotic genus Lactobacillus modulate immune responses. Front. Pharmacol. 13:1042189. doi: 10.3389/fphar.2022.1042189, 36353491 PMC9638459

[ref31] Sadanov A. Alimzhanova M. Ismailova E. Shemshura O. Ashimuly K. Molzhigitova A. . (2023). Antagonistic and protective activity of *Lactobacillus plantarum* strain 17 M against *E. amylovora*. World J. Microbiol. Biotechnol. 39:314. doi: 10.1007/s11274-023-03765-3, 37733156

[ref32] Seemann T. (2014). Prokka: rapid prokaryotic genome annotation. Bioinformatics 30, 2068–2069. doi: 10.1093/bioinformatics/btu153, 24642063

[ref33] Sun X. Jiao C. Schwaninger H. Chao C. T. Ma Y. Duan N. . (2020). Phased diploid genome assemblies and pan-genomes provide insights into the genetic history of apple domestication. Nat. Genet. 52, 1423–1432. doi: 10.1038/s41588-020-00723-9 33139952 PMC7728601

[ref34] Torres-Maravilla E. Reyes-Pavón D. Benítez-Cabello A. González-Vázquez R. Ramírez-Chamorro L. M. Langella P. . (2022). Strategies for the identification and assessment of bacterial strains with specific probiotic traits. Microorganisms 10:1389. doi: 10.3390/microorganisms10071389, 35889107 PMC9323131

[ref35] Yao M. Zhang M. Lai T. Yang Z. (2022). Characterization and in vitro fecal microbiota regulatory activity of a low-molecular-weight exopolysaccharide produced by *Lactiplantibacillus plantarum* NMGL2. Foods 11:393. doi: 10.3390/foods11030393, 35159543 PMC8834501

[ref36] Yu A. O. Leveau J. H. J. Marco M. L. (2020). Abundance, diversity and plant-specific adaptations of plant-associated lactic acid bacteria. Environ. Microbiol. Rep. 12, 16–29. doi: 10.1111/1758-2229.12794, 31573142

[ref37] Zhang L. Kulyar M. F. Niu T. Yang S. Chen W. (2024). Comparative genomics of Limosilactobacillus reuteri YLR001 reveals genetic diversity and probiotic properties. Microorganisms 12:1636. doi: 10.3390/microorganisms12081636, 39203478 PMC11356486

[ref38] Zhang X. Liu X. Yang F. Chen L. (2018). Pan-genome analysis links the hereditary variation of *Leptospirillum ferriphilum* with its evolutionary adaptation. Front. Microbiol. 9:577. doi: 10.3389/fmicb.2018.00577, 29636744 PMC5880901

[ref39] Zhang Z. Niu H. Qiu Q. Guo D. Wan X. Yang Q. . (2025). Advancements in *Lactiplantibacillus plantarum*: probiotic characteristics, gene editing technologies and applications. Crit. Rev. Food Sci. Nutr. 65, 6623–6644. doi: 10.1080/10408398.2024.2448562 39745813

[ref40] Zhao H. Ali U. Ren Q. Yao M. Lai T. Naz S. . (2023). Integrated metabolomic analysis of *Lactiplantibacillus plantarum* NMGL2 reveals its survival and response to combinational cold and acidic conditions during storage of fermented milk. Food Biosci. 54:102833. doi: 10.1016/j.fbio.2023.102833

[ref41] Zheng J. Wittouck S. Salvetti E. Franz C. M. A. P. Harris H. M. B. Mattarelli P. . (2020). A taxonomic note on the genus Lactobacillus: description of 23 novel genera, emended description of the genus Lactobacillus Beijerinck 1901, and union of Lactobacillaceae and Leuconostocaceae. Int. J. Syst. Evol. Microbiol. 70, 2782–2858. doi: 10.1099/ijsem.0.004107 32293557

